# Simultaneous acquisition of maternal and dual‐fetal electrocardiography with maternal haemodynamic monitoring in anaesthetized pregnant rats

**DOI:** 10.1113/EP094260

**Published:** 2026-10-28

**Authors:** Sarah Pribil Pardun, Spencer Duff, Payton Ward, Auspy Li, Lie Gao

**Affiliations:** ^1^ Department of Anesthesiology University of Nebraska Medical Center Omaha Nebraska USA

**Keywords:** autonomic regulation, electrocardiography, fetus, heart rate variability, haemodynamics, maternal–fetal cardiovascular dynamics, pregnancy, rat

## Abstract

Experimental approaches that acquire maternal haemodynamics together with direct cardiac signals from more than one fetus remain limited. We developed a standardized in vivo method for simultaneous acquisition of maternal ECG, maternal arterial blood pressure and ECGs from two fetuses in anaesthetized late‐gestation Sprague–Dawley rats. The platform combines custom‐fabricated needle electrodes with conventional cardiovascular instrumentation and PowerLab/LabChart acquisition. Stable, analysable maternal arterial blood pressure, maternal ECG and dual‐fetal ECG were maintained for ≤5 h. The recordings supported beat‐to‐beat ECG interval and heart rate variability analyses. Fetuses had lower heart rates and longer R–R intervals than dams, and fetal root mean square of successive differences (RMSSD), high‐frequency power and standard deviation of RR intervals (SDRR) were lower than maternal values. As proof of recording responsiveness, maternal noradrenaline and verapamil administration produced readily resolved maternal haemodynamic changes together with time‐aligned fetal heart rate responses. This protocol does not by itself demonstrate maternal–fetal cardiac coupling; rather, it provides synchronized high‐resolution signals suitable for future analyses of dynamic maternal–fetal cardiovascular relationships and pharmacological responses.

## Abstract (highlights)

- **What is the central question of this study?**
  Can a reproducible in vivo preparation provide prolonged, simultaneous maternal arterial pressure, maternal ECG and direct ECG recordings from two fetuses in the same pregnant rat?
- **What is the main finding and its importance?**
  The method produced stable maternal blood pressure, maternal ECG and dual‐fetal ECG recordings for ≤5 h and supported ECG/heart rate variability analysis and acute pharmacological testing. Its principal advance is the integration of continuous maternal haemodynamics with direct, simultaneous ECG from two fetuses, providing synchronized signals for integrative maternal–fetal cardiovascular physiology without claiming that concurrent responses alone establish cardiac coupling.

## 1 INTRODUCTION

Maternal and fetal cardiovascular states change continuously during pregnancy and are influenced by maternal haemodynamics, autonomic activity, placental function, endocrine and metabolic signalling, and pharmacological exposure (Bowman et al., 2021; Kramer et al., 2023). Simultaneous maternal and fetal cardiac recordings can therefore provide an important physiological framework for examining time‐aligned cardiovascular responses. Maternal–fetal cardiac coupling has been described using dynamic and beat‐to‐beat approaches in humans and animal models, but the presence and direction of coupling require dedicated analyses of covariation, phase relationships or other time‐resolved metrics rather than simple comparison of static heart rate or heart rate variability (HRV) values (Khandoker et al., 2018, 2019, 2022; Nichting et al., 2023).

Direct fetal ECG has important advantages for resolving fetal rhythm and conduction, whereas maternal arterial pressure provides a simultaneous measure of the maternal haemodynamic perturbation to which the fetuses are exposed. Prior work has established the feasibility of simultaneous maternal and fetal ECG in mice (Khandoker et al., 2018, 2022). Recent analytical work has also emphasized time‐resolved autonomic analysis in rats rather than relying only on time‐averaged HRV indices (Candia‐Rivera et al., 2026). However, a standardized rat preparation that combines continuous maternal arterial pressure, maternal ECG and direct ECG from two fetuses within the same pregnancy has not been well described.

Recording from two fetuses is methodologically useful because littermates share the same maternal perturbation while permitting simultaneous assessment of two fetal signals. This design does not, by itself, establish maternal–fetal or inter‐fetal coupling, but it creates the synchronized data streams required for future analyses of dynamic covariation, response latency, phase relationships and fetus‐to‐fetus heterogeneity in common maternal conditions.

Pharmacological perturbation provides a practical means of demonstrating that the acquisition system resolves rapid maternal cardiovascular changes together with fetal cardiac responses. Such experiments should be interpreted as functional validation of recording responsiveness rather than direct evidence of the mechanism linking maternal and fetal responses, because transplacental drug transfer, uteroplacental haemodynamics and secondary neurohumoral pathways might all contribute.

Accordingly, we developed a reproducible Methods and Techniques protocol for simultaneous maternal ECG, dual‐fetal ECG and maternal arterial blood pressure (BP) recording in anaesthetized late‐gestation rats. The method combines custom needle electrodes with standard cardiovascular instrumentation and commercially available acquisition software, permits prolonged recording and supports beat‐to‐beat ECG and HRV analyses.

We describe the instrumentation, electrode placement, troubleshooting and analysis workflow and use noradrenaline (NA) and verapamil as proof‐of‐function perturbations. The primary contribution is methodological; that is, synchronized acquisition of maternal haemodynamics and direct ECG signals from two fetuses, providing a platform for future mechanistic and dynamic studies of maternal–fetal cardiovascular physiology.

## 2 MATERIALS AND METHODS

### 2.1 Ethical approval

All animal procedures were carried out in accordance with the institutional regulation approved by the Animal Care and Use Committee of the University of Nebraska Medical Center (UNMC‐IACUC Protocol #24‐012‐054; approved 12 December 2024) and complied with the animal experiment policies of *Experimental Physiology*. This study also adhered to the NIH *Guide for the Care and Use of Laboratory Animals* (8th edn, revised 2011) and the Animals in Research: Reporting In Vivo Experiments (ARRIVE) 2.0 guidelines. Efforts were made to minimize animal use while obtaining the repeated maternal and dual‐fetal recordings required for methodological validation.

### 2.2 Experimental animals

Six timed‐pregnant Sprague–Dawley rats (gestational day 18–20) were purchased from Charles River Laboratories (Wilmington, MA, USA) and housed in the University of Nebraska Medical Center animal facility under a 12 h–12 h light–dark cycle with ad libitum access to water and standard chow. Experiments were performed under isoflurane general anaesthesia (2% in oxygen for induction by chamber; 1.5%–2.0% in oxygen for maintenance by nose cone). At the end of the terminal experiment, animals were killed via inhalation of an overdose of isoflurane, followed by bilateral thoracotomy to confirm death.

### 2.3 General experimental design

The platform was designed for synchronized recording of maternal arterial BP, maternal ECG and direct ECG from two fetuses during late gestation. After induction of anaesthesia and stabilization, the maternal femoral artery was instrumented for continuous pressure recording and the femoral vein for drug administration. Maternal ECG electrodes were placed subcutaneously, and one accessible fetus from each uterine horn was selected for direct bipolar fetal ECG electrode placement. Following signal stabilization, all four channels were recorded concurrently for baseline characterization, prolonged‐stability testing and pharmacological proof‐of‐function experiments. ECG and HRV analyses were performed offline in LabChart.

### 2.4 Anaesthesia and physiological monitoring

A Vapomatic veterinary vaporizer (A.M. Bickford, Wales Center, NY, USA) was used for anaesthetic delivery, and temperature was maintained with a TCAT‐2LV Animal Temperature Controller (Physitemp, Clifton, NJ, USA). Rats were anaesthetized with isoflurane delivered in oxygen via an induction chamber (2% isoflurane), then transferred to a heated surgical table in the supine position. Anaesthesia was maintained through a nose cone with 1.5%–2.0% isoflurane. Ophthalmic ointment was applied to prevent corneal drying. Adequate depth of anaesthesia was confirmed by the absence of withdrawal and corneal reflexes. Respiratory rate and body temperature were monitored continuously throughout the experiment. A temperature‐controlled heating pad was set to 32°C, and additional heat support was provided with a heat lamp as needed to maintain maternal and fetal thermal stability. Hypothermia was avoided because it reduced ECG signal stability and blunted cardiovascular responsiveness.

### 2.5 Data‐acquisition hardware and recording set‐up

Signals were acquired using a PowerLab 16/30 data‐acquisition system (ML880; ADInstruments, Colorado Springs, CO, USA) coupled to three Grass P55 A.C. preamplifiers (Natus Medical, Middleton, WI, USA; Figure 1a). Maternal arterial pressure was monitored through a Millar Pressure Control Unit (PCU‐2000; Millar, Pearland, TX, USA) using a Millar Mikro‐Tip pressure catheter (SPR‐407; ADInstruments). The PowerLab was operated at a sampling rate of 1 kHz. ECG signals were amplified and band‐pass filtered at 30 Hz (high‐pass) and 3 kHz (low‐pass). Separate acquisition channels were used for maternal BP, maternal ECG, fetus A ECG and fetus B ECG. Custom needle‐style electrodes (Figure 1b) were fabricated using platinum–iridium alloy wire (Pt90/Ir10; 0.0159‐inch diameter for maternal ECG and 0.007‐inch diameter for fetal ECG; Sigmund Cohn Corp., Mount Vernon, NY, USA) as the electrode core. The alloy wires were soldered to PVC‐insulated copper leads (26 AWG; Calmont Wire & Cable, Santa Ana, CA, USA), and the junctions were insulated with heat‐shrink tubing (1/16–1/32 inch; Gardner Bender, Milwaukee, WI, USA), which served as the electrode shaft. To minimize environmental electrical interference, recordings were performed on vibration‐controlled workstations with Faraday cages (1200 Series Workstation; Kinetic Systems, Boston, MA, USA; Figure 1c). All equipment and animals were properly earthed before each experiment to minimize electrical noise.

**FIGURE 1:**
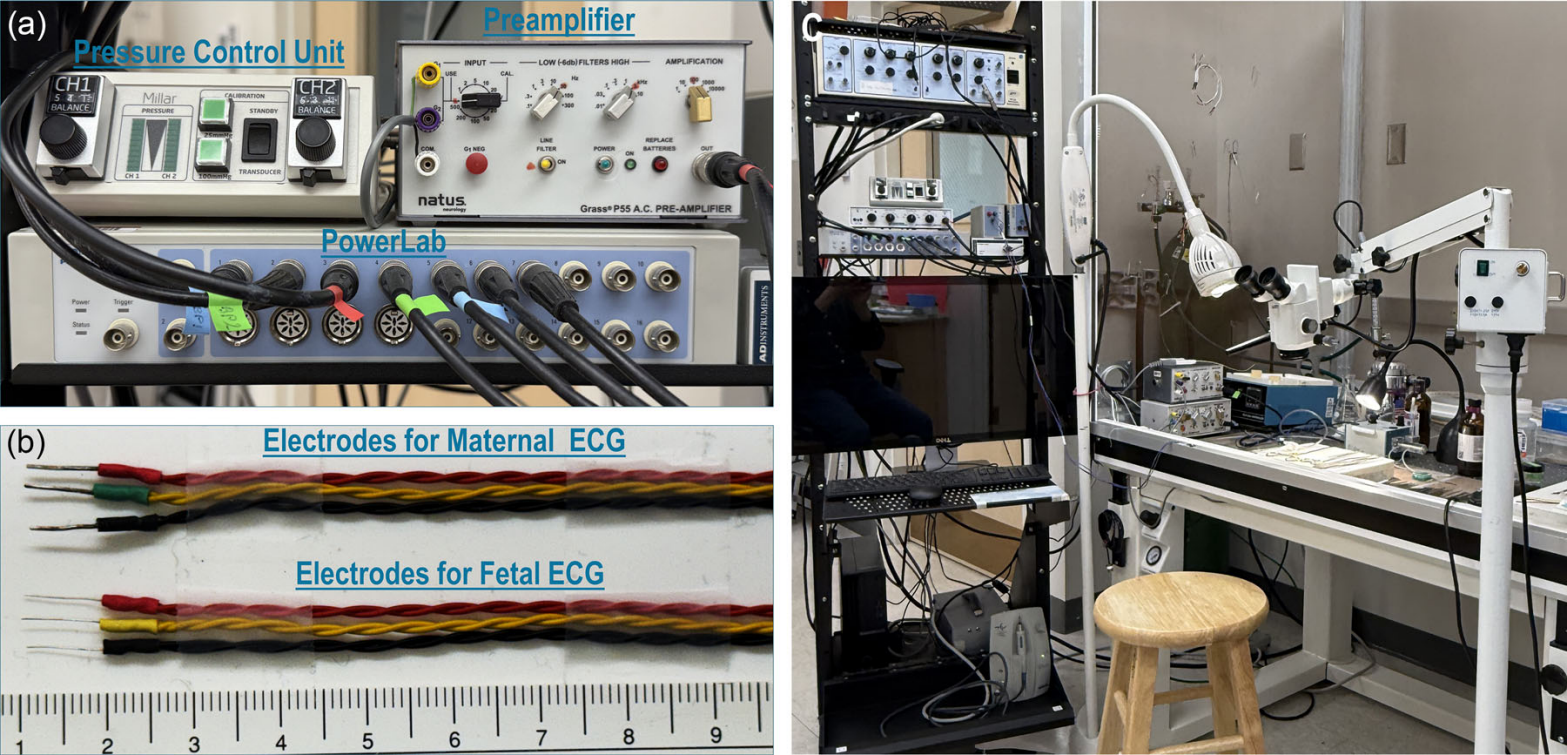
Main equipment and custom‐built electrodes used for simultaneous maternal and dual‐fetal electrocardiography recording. (a) Main recording hardware, including the pressure control unit, preamplifier and PowerLab data‐acquisition system. (b) Custom‐built needle‐style electrodes used for maternal ECG and fetal ECG recordings. (c) The whole workstation with vibration control desks and Faraday cages.

### 2.6 Data‐acquisition software and analyses

The software interface during recording is shown in Figure 2. Figure 2a shows the operation interface of LabChart (MLS060/8; ADInstruments) and its sub‐modules, including the blood pressure module (Figure 2a1, red box; MLS370/8; ADInstruments), ECG analysis module (Figure 2a2, green box; MLS360/8; ADinstruments) and HRV module (Figure 2a2, blue box; MLS310/8; ADinstruments). Figure 2b displays four simultaneous recording channels: maternal BP, maternal ECG, fetus A ECG and fetus B ECG. Blue and green circles indicate detected beat markers and R waves used for RR interval‐based analysis in fetus A and fetus B, respectively.

**FIGURE 2:**
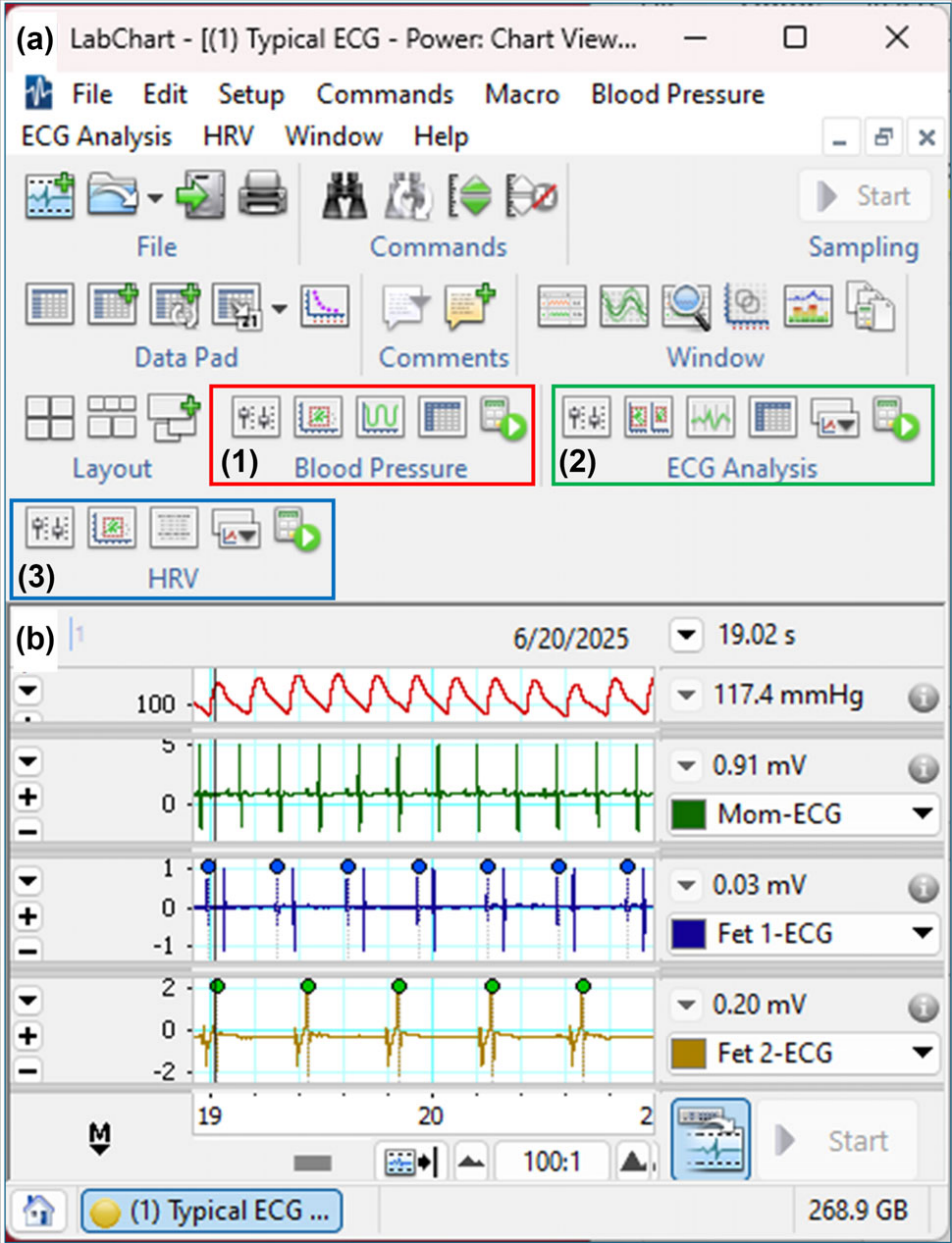
LabChart Software interface used for maternal blood pressure, maternal ECG and dual‐fetal ECG acquisition and analysis. (a) LabChart interface, showing the blood pressure module (1), ECG analysis module (2) and HRV module (3). (b) Representative recording channels, showing maternal arterial blood pressure, maternal ECG, fetus‐A ECG and fetus‐B ECG.

ECG intervals were measured with the LabChart ECG analysis module, and HRV was calculated from verified RR intervals with the HRV module. Automated beat detection was visually inspected and corrected when necessary. The ECG electrode implacement is illustrated in Figure 3, and a full recording of ECG is shown in Figure 4. The analysis workflow is summarized below and illustrated in Figures 5 and 6.

**FIGURE 3:**
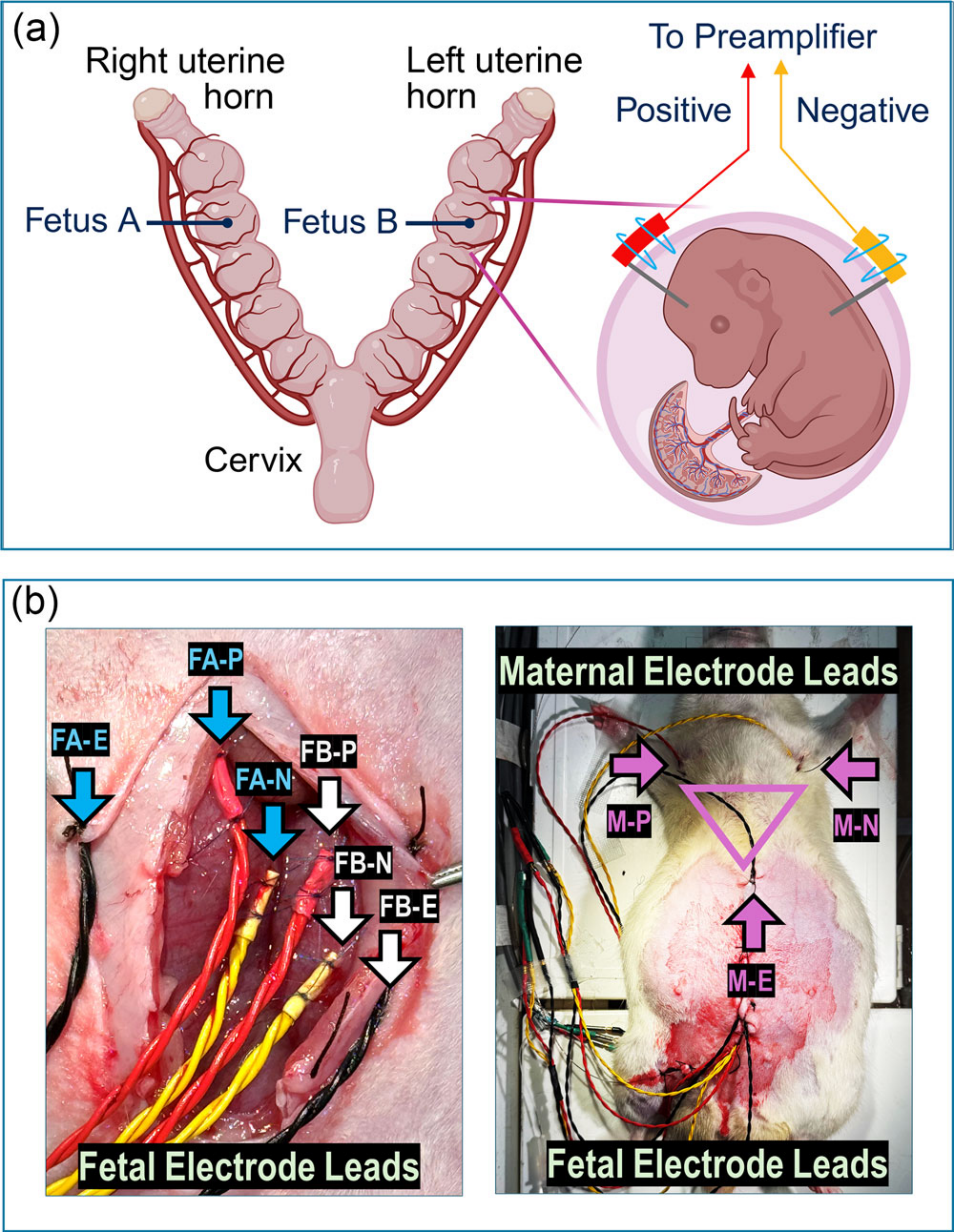
Electrode placement for simultaneous maternal and dual‐fetal ECG recording in pregnant rats. (a) Schematic illustration of fetal electrode placement in two fetuses, one selected from each uterine horn. (b) Representative surgical images, showing electrode placement and lead routing. Abbreviations: E, earth; FA, fetus A; FB, fetus B; M, maternal; N, negative; P, positive.

**FIGURE 4:**
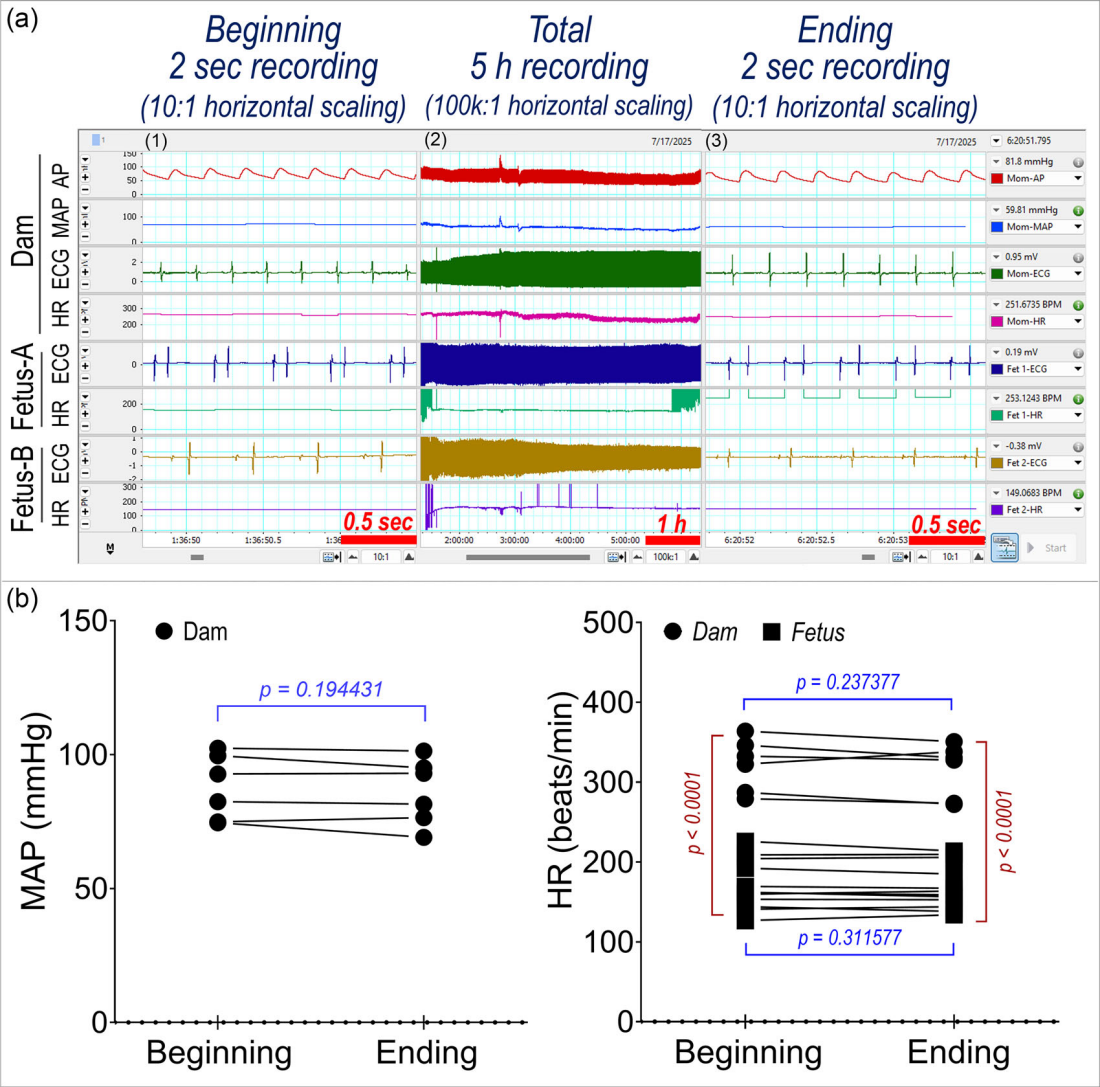
Stability of maternal and dual‐fetal cardiovascular recordings during a 5 h acquisition period. (a) Representative recording of maternal arterial blood pressure (AP), maternal ECG and dual‐fetal ECG obtained from one dam and two fetuses. (a1) Expanded traces at 5:1 horizontal scaling for a 2 s recording at the beginning. (a2) Compressed traces at 100 000:1 horizontal scaling for a 5 h recording. (a3) Expanded traces at 5:1 horizontal scaling for a 2 s recording at the ending. (b) Group data showing mean arterial pressure (MAP) and HR of dams and fetuses at the first 30 min and last 30 min of recordings. *n* = 6 for dams and *n* = 12 for fetuses. HR was derived from the RR interval of the ECG.

**FIGURE 5:**
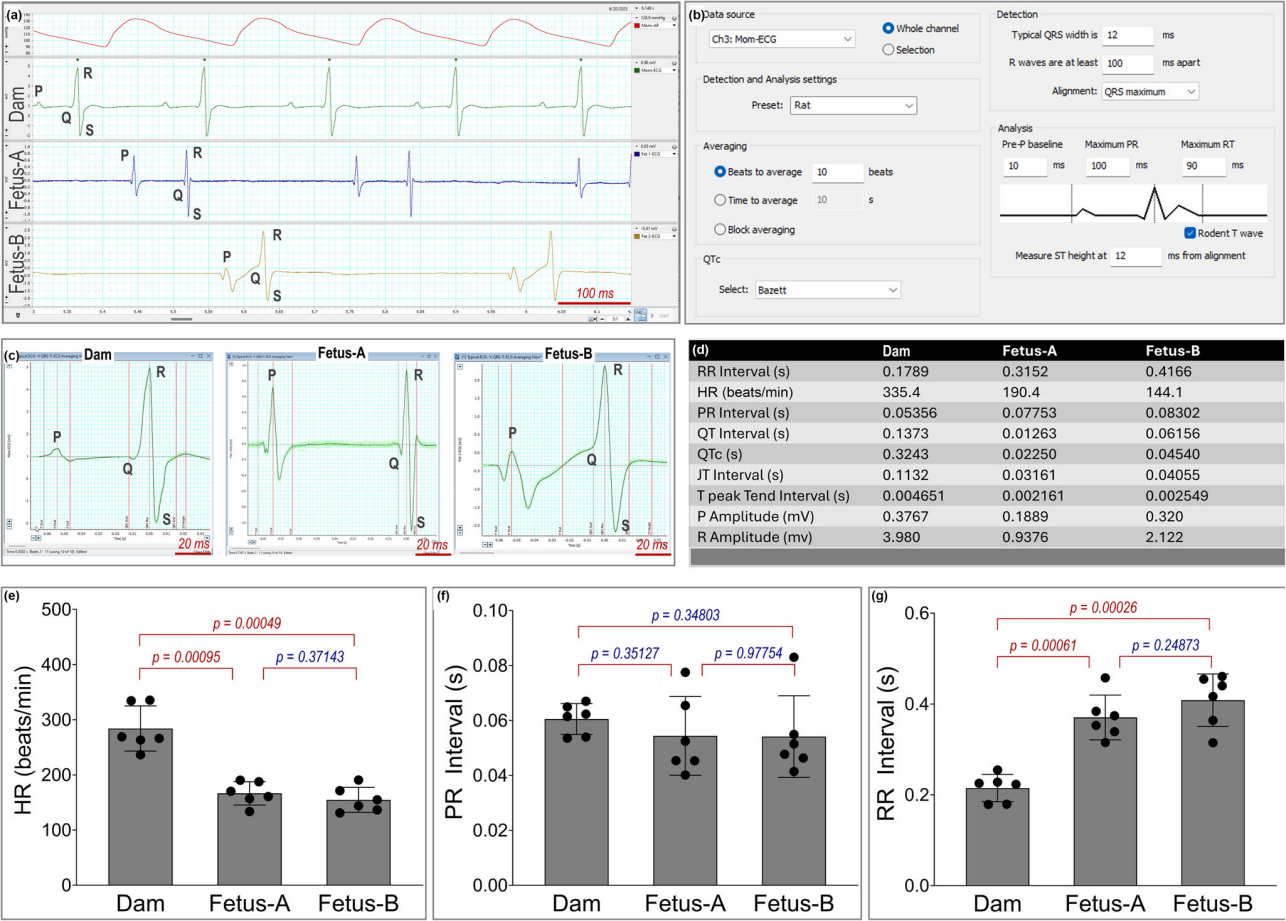
ECG waveform analysis in dams and fetuses. (a) Representative ECG recordings showing the major waveform components, including the P wave and QRS complex, from the dam and two fetuses. (b) ECG analysis settings window. (c) ECG averaging view. (d) Representative ECG parameters obtained from one dam and her two fetuses. (e–g) Group data for heart rate, PR interval and RR interval. *n* = 6 dams or fetuses per group. Fetus A was selected from the right uterine horn and fetus B from the left uterine horn.

**FIGURE 6:**
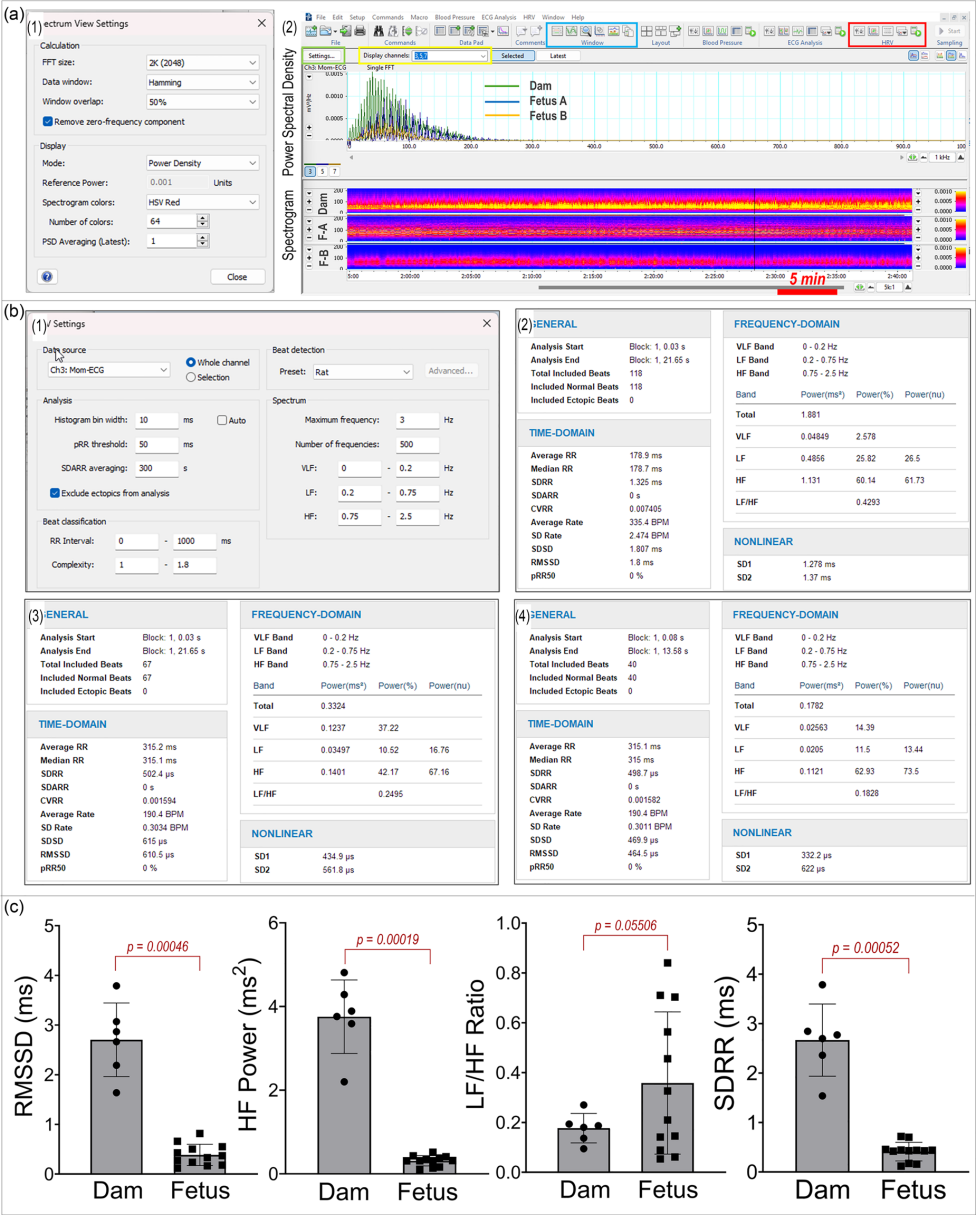
Power spectral density and heart rate variability analysis of maternal and fetal ECG signals. (a) Representative power spectral density/spectrogram settings (a1) and output (a2). (b) HRV settings window (b1) and representative report views from the dam (b2), fetus A (b3) and fetus B (b4). (c) Group data for representative HRV parameters, including RMSSD, HF power, LF/HF ratio and SDRR, obtained from maternal and fetal ECG. ECG: electrocardiogram; HF: high frequency power; HRV: heart rate variability; LF: low frequency power; RMSSD: root mean square of successive differences; SDRR: standard deviation of RR intervals.

### 2.7 Statistical analysis

Maternal data were obtained from six dams. Two fetal recordings were obtained from each dam (12 fetal recordings from six pregnancies). Statistical analyses were performed in Prism 8 (GraphPad Software). The analysis used the comparisons shown in the original figures: paired beginning versus ending comparisons (Figure 4), comparisons among dam, fetus A and fetus B (Figure 5), dam versus fetus comparisons (Figure 6) and planned within‐recording comparisons before, during and after drug administration (Figures 7 and 8). Data are presented as the mean ± SD with individual observations shown in the figures. Exact *P*‐values are reported in the figures and in the corresponding Results text; *P* < 0.0001 is used only when appropriate. Because two fetal recordings originated from each pregnancy, their shared maternal origin is stated explicitly throughout to avoid interpretation of 12 fetal recordings as 12 independent pregnancies.

**FIGURE 7:**
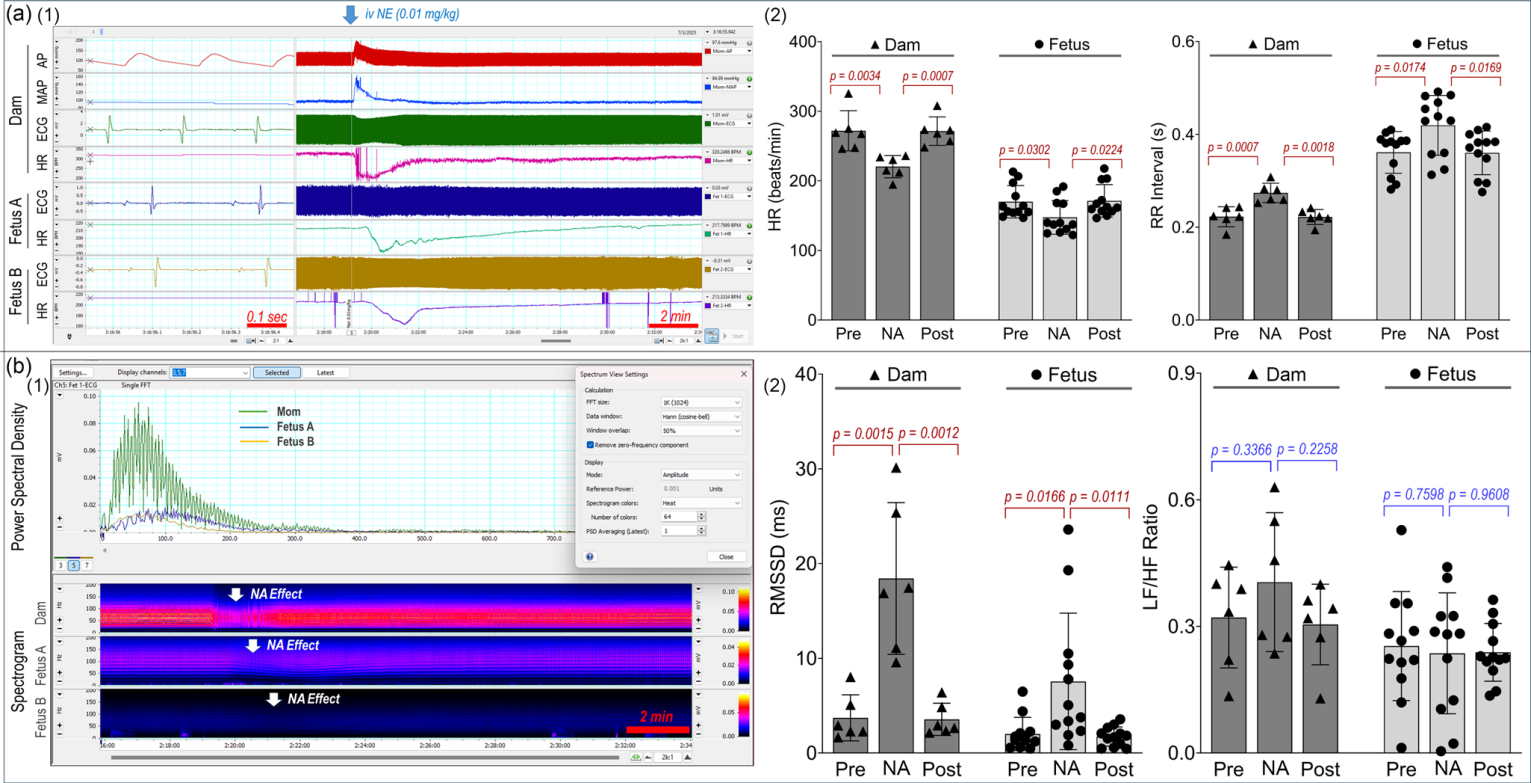
Effects of maternal NA administration on maternal and fetal cardiovascular signals. (a) Representative recording (a1) of maternal arterial blood pressure, maternal ECG, and dual‐fetal ECG after intravenous NA (0.01 mg kg^−^^1^), and group data (a2) showing HR and RR interval responses in dams and fetuses. (b) Representative power spectral density and spectrogram output (b1) and group data (b2) showing RMSSD and LF/HF ratio before, during and after NA administration. HR was derived from the RR interval of the ECG. *n* = 6 dams and *n* = 12 fetuses. Data are means ± SD. Abbreviations: HR, heart rate; NA, noradrenaline; NA effect, maximal NA effect; Post, after NA injection; Pre, before NA injection. LF/HF: low frequency power/high frequency power; RMSSD: root mean square of successive differences.

**FIGURE 8:**
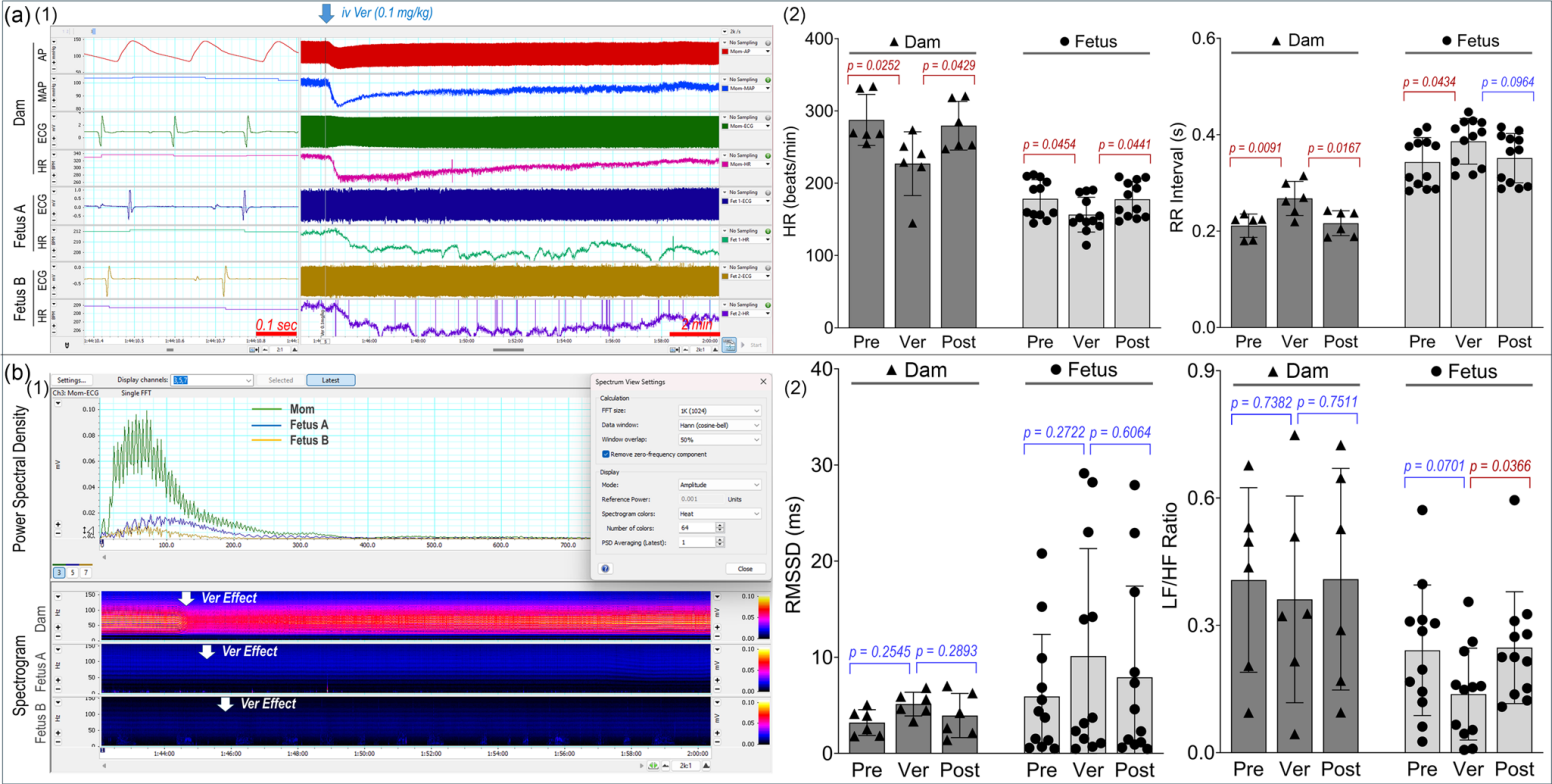
Effects of maternal verapamil administration on maternal and fetal cardiovascular signals. (a) Representative recording (a1) of maternal arterial blood pressure, maternal ECG and dual‐fetal ECG after intravenous Ver (0.1 mg kg^−^^1^), and group data (a2) showing HR and RR interval responses in dams and fetuses. (b) Representative power spectral density and spectrogram output (b1) and group data (b2) showing RMSSD and LF/HF ratio before, during, and after verapamil administration. HR was derived from RR interval of ECG. *n* = 6 dams and *n* = 12 fetuses. Data are means ± SD. Abbreviations: HR, heart rate; Post, after Ver injection; Pre, before Ver injection; Ver, verapamil; Ver effect, maximal Ver effect.

### 2.8 Experimental procedures

#### 2.8.1 Preoperative preparation and equipment set‐up

The PowerLab, Grass preamplifiers and the LabChart computer were switched on before surgery. Channels were configured for maternal BP, maternal ECG, fetus A ECG and fetus B ECG; sampling was set to 1 kHz and the ECG preamplifier band‐pass to 30 Hz (high‐pass) and 3 kHz (low‐pass). The surgical table was prepared with a temperature‐controlled heating pad set to 32°C; a higher pad setting was avoided to reduce maternal hyperthermia. Surgical instruments were sterilized according to institutional practice.

#### 2.8.2 Anaesthesia and maternal physiological stabilization

General anaesthesia was induced with 2% isoflurane in oxygen in an induction chamber and maintained at 1.5%–2.0% through a nose cone after the rat was placed supine on the heated table. Ophthalmic ointment was applied, anaesthetic depth was confirmed by absent withdrawal and corneal reflexes, and respiratory rate and body temperature were monitored continuously. Maternal abdominal and fetal temperature were protected with the heating pad and, when necessary, a heat lamp because fetal cooling produced bradycardia and reduced signal stability.

#### 2.8.3 Maternal vascular instrumentation

A right inguinal incision was made, and the femoral artery was isolated for insertion of a Millar pressure catheter. After a stable pulsatile pressure waveform was confirmed, the catheter was secured. The right femoral vein was then cannulated and secured for intravenous drug administration. Vascular catheters were flushed with heparinized saline as needed.

#### 2.8.4 Exposure of gravid uterus and fetal selection

A midline abdominal incision ∼5 cm in length was made to expose the gravid uterus. The uterine horns were gently exteriorized, kept moist with warm sterile saline, and manipulated as little as possible to preserve uteroplacental perfusion. One accessible fetus from each uterine horn was selected to maximize spatial separation; in practice, the second fetus from the distal end of each horn usually provided convenient access, with the third fetus used if signal stability was inadequate.

#### 2.8.5 Fetal ECG electrode implantation

Figure 3 illustrates fetal electrode placement and lead routing. For each selected fetus, the anterior fontanelle and upper lumbar region were identified through the uterine wall. The positive electrode core, bent ∼90° relative to its shaft, was inserted through the anterior fontanelle into the forebrain, and the shaft was secured to the uterine surface with two 6–0 non‐absorbable polypropylene sutures. Deep posterior insertion was avoided to prevent brainstem injury. The negative electrode was inserted through the upper lumbar region into the upper abdominal cavity and secured in a similar manner; thoracic penetration was avoided. The procedure was repeated for the second fetus. Electrode cores were kept fully covered by the uterine wall; membrane disruption and amniotic fluid leakage were minimized, and electrode shafts were firmly fixed to maintain stable prolonged recordings. An earth electrode was placed subcutaneously in the maternal abdominal wall.

#### 2.8.6 Abdominal closure and signal stabilization

The uterine horns were returned gently to their original intra‐abdominal positions without torsion or lead tension. Maternal and fetal ECG signal quality was verified; the abdominal incision was closed in layers with 3–0 non‐absorbable polypropylene sutures, and the preparation was allowed to stabilize for 10–15 min before baseline recording.

#### 2.8.7 Baseline ECG and haemodynamic recording

Maternal ECG, dual‐fetal ECG and maternal arterial pressure were recorded continuously for ≥30 min before intervention. Waveforms were inspected for stable QRS detection and acceptable signal‐to‐noise ratio, and the baseline period was kept uninterrupted for within‐recording comparisons.

#### 2.8.8 Pharmacological interventions

For proof‐of‐function testing, noradrenaline (0.01 mg kg^−1^, i.v.) was administered through the maternal femoral vein and signals were recorded for 25 min, followed by ∼30 min of recovery. Verapamil (0.1 mg kg^−1^, i.v.) was then administered and recording continued for a further 25 min. These agents were selected to produce rapid, readily measurable but mechanistically distinct maternal cardiovascular perturbations; the experiments were used to demonstrate acquisition responsiveness rather than to establish a mechanism of maternal–fetal coupling.

#### 2.8.9 Prolonged recording and termination

Recordings were continued for ≤5 h to test the stability of the preparation. During prolonged experiments, 0.9% sodium chloride was infused through the femoral venous catheter at 1 mL kg^−1^ h^−1^ to reduce dehydration. The 5 h duration represents a stress test of preparation stability; 60–90 min recording windows were generally sufficient for acute pharmacological experiments. At the end of the experiment, animals were euthanized under deep anaesthesia using the approved method stated in section 2.1.

### 2.9 Troubleshooting

Common technical problems, likely causes and corrective actions are summarized in Table 1.

**TABLE 1:** Troubleshooting guide for simultaneous maternal and dual‐fetal cardiovascular recording.

	Problem	Possible reason	Solution
1	Excessive baseline drift	Incorrect filter settings	Confirm high‐pass at 30 Hz and low‐pass at 3 kHz
2	High electrical noise across channels	Improper earthing	Ensure all devices, animals, workstations and Faraday cages share common earth; verify shielding of electrode cables
3	Dampened or flat arterial pressure trace	Catheter sensor blocked by blood clots	Flush the artery with heparinized saline
4	Unstable arterial pressure signal	Catheter displacement	Reposition and resecure catheter; confirm pulsatile waveform
5	Unstable fetal ECG	Electrode displacement	Re‐secure electrode shaft to uterine wall with additional sutures
6	Very low fetal ECG amplitude	Incomplete electrode core insertion	Confirm full insertion into brain and abdominal cavity
7	Abrupt fetal bradycardia	Brainstem injury	Avoid deep posterior insertion; ensure insertion is limited to the forebrain
8	Sudden loss of fetal ECG	Thoracic injury or haemorrhage	Avoid chest cavity penetration; insert into upper abdominal region only
9	Increasing noise over time	Amniotic fluid leakage	Minimize membrane disruption; avoid excessive uterine manipulation
10	Noisy ECG signal	Poor earthing or loose connector	Check common earth; inspect cable connections and shielding
11	Maternal ECG contaminating fetal channel	Earth electrode improperly placed	Confirm subcutaneous maternal earth placement and separation of leads
12	Loss of fetal signal during repositioning	Uterine torsion or tension on electrodes	Reposition uterine horns gently; avoid twisting electrode shafts
13	Progressive fetal bradycardia	Fetal hypothermia	Reassess abdominal temperature; use heat lamp if necessary
14	Progressive maternal hypotension	Excess anaesthetic depth	Reduce isoflurane concentration; ensure adequate oxygen delivery
15	No pharmacological response	Drug degradation or catheter blockage	Prepare fresh drug; confirm intravenous patency
16	Signal deterioration during prolonged recording	Dehydration	Administer saline (1 mL kg^−1^ h^−1^) via femoral vein catheter

## 3 RESULTS

### 3.1 Stability of the recording system

We evaluated preparation stability during a continuous 5 h acquisition (Figure 4). Representative traces (Figure 4a) showed sustained maternal arterial pressure, maternal ECG and dual‐fetal ECG signal quality from the beginning to the end of recording. Figure 4a1 presents expanded traces at the beginning of 5 h recording, demonstrating individual arterial pressure pulses and ECG waveforms across seven consecutive maternal cardiac cycles. Figure 4a2 displays the compressed full‐length recording, highlighting sustained signal quality and stability of maternal BP and ECG signals and dual‐fetal ECG signals, throughout the 5 h period. Figure 4a3 presents expanded traces at the end of 5 h recording, demonstrating no substantial alteration of the arterial pressure pulses and ECG waveforms compared with those at the beginning. Across six dams and 12 fetal recordings from six pregnancies (Figure 4b), maternal MAP did not differ between the first and last 30 min (*P* = 0.194431), maternal heart rate (HR) did not differ (*P* = 0.237377), and fetal HR did not differ (*P* = 0.311577). Fetal HR remained lower than maternal HR at both time points (*P* < 0.0001). These findings support stable haemodynamic and electrophysiological acquisition over the tested interval.

### 3.2 ECG waveform and interval analysis

Figure 5 shows representative maternal, fetus A and fetus B ECGs and the LabChart ECG‐analysis workflow. The P waves and QRS complexes were identifiable in all channels, and fetal ECG cycles were independent of the maternal cardiac cycle (Figure 5a, c). Compared with dams, heart rate was lower in fetus A (*P* = 0.000950) and fetus B (*P* = 0.000490), whereas fetus A and fetus B did not differ (*P* = 0.371). The RR interval was longer in fetus A (*P* = 0.000610) and fetus B (*P* = 0.000260) than in dams, with no difference between fetal positions (*P* = 0.249). The PR interval did not differ between the dam and fetus A (*P* = 0.351), the dam and fetus‐B (*P* = 0.348) or fetus A and fetus B (*P* = 0.978). These analyses demonstrate that the recorded signals support standard interval‐based ECG measurements.

#### 3.2.1 ECG analysis workflow

Saved ECG channels were analysed with the LabChart ECG analysis module using the rat preset. Detection was verified visually in the ECG averaging view; the maximum PR interval was increased to 100 ms when required for fetal morphology, and fiducial points were corrected manually when automated placement was inaccurate. ECG parameters were then exported from table view (Figure 5d).

### 3.3 Heart rate variability and power spectral analysis

HRV was calculated from verified RR intervals using the LabChart HRV module (Figure 6). Figure 6a shows the spectrum view settings (Figure 6a1) and a representative power spectrum and spectrogram output (Figure 6a2). Figure 6b displays the HRV settings window (Figure 6b1) and representative report views for the dam (Figure 6b2), fetus A (Figure 6b3) and fetus B (Figure 6b4). Figure 6c summarizes group data for four representative HRV parameters derived from the time‐domain and frequency‐domain analysis, including root mean square of successive differences (RMSSD), standard deviation of RR intervals (SDRR), high‐frequency (HF) power and the low‐frequency/high‐frequency (LF/HF) ratio. Compared with dams, fetuses had lower RMSSD (*P* = 0.000460), lower HF power (*P* = 0.000190) and lower SDRR (*P* = 0.000520). The LF/HF ratio did not differ significantly between groups (*P* = 0.0551). These static HRV indices characterize the signals but are not presented as evidence of dynamic maternal–fetal cardiac coupling.

#### 3.3.1 Heart rate variability analysis workflow

After ECG beat detection, the corresponding channels were analysed in the HRV module with the rat preset and visually inspected. Spectrum view was used to display power spectral density and spectrograms, and report view was used to export the selected time‐ and frequency‐domain HRV measures.

### 3.4 Effects of cardiovascular drugs

To test whether the recording platform resolves acute maternal perturbations and concurrent fetal cardiac responses, we examined noradrenaline (NA) and verapamil (Ver) as proof‐of‐function interventions.

#### 3.4.1 Noradrenaline responses

Figure 7 summarizes maternal and fetal responses to an intravenous maternal bolus of NA (0.01 mg kg^−1^), including maternal BP, maternal/fetal ECG and HRV‐derived measures. NA produced an immediate maternal pressor response with bradycardia and a delayed decrease in fetal HR. Maternal HR decreased from pre‐injection to the maximal NA effect (*P* = 0.00340) and recovered thereafter (NA vs. post, *P* = 0.000700); fetal HR changed in a similar manner (pre vs. NA, *P* = 0.00320; NA vs. post, *P* = 0.00260). The RR interval increased in dams (pre vs. NA, *P* = 0.000700; NA vs. post, *P* = 0.00140) and fetuses (*P* = 0.0174 and *P* = 0.0169, respectively). The RMSSD increased at the maximal NA response in dams (*P* = 0.00150) and fetuses (*P* = 0.0106) and returned towards baseline (NA vs. post: dams, *P* = 0.00120; fetuses, *P* = 0.0111). The LF/HF ratio did not differ significantly in dams (*P* = 0.337 and *P* = 0.226) or fetuses (*P* = 0.760 and *P* = 0.0608) for the displayed planned comparisons.

#### 3.4.2 Verapamil responses

Figure 8 summarizes maternal and fetal responses to an intravenous maternal bolus of Ver (0.1 mg kg^−1^), including maternal BP, maternal/fetal ECG and HRV‐derived measures. Ver produced maternal hypotension and bradycardia together with fetal bradycardia. Maternal HR decreased from pre‐injection to the maximal Ver effect (*P* = 0.00520) and recovered thereafter (*P* = 0.0429); fetal HR showed corresponding changes (*P* = 0.0454 and *P* = 0.0441). Maternal RR interval increased at the maximal effect (*P* = 0.000100) and decreased during recovery (*P* = 0.00160). Fetal RR interval increased from pre‐injection to maximal effect (*P* = 0.0434), whereas the comparison of maximal effect versus recovery was not significant (*P* = 0.0964). The RMSSD did not change significantly in dams (*P* = 0.255 and *P* = 0.289) or fetuses (*P* = 0.272 and *P* = 0.606). The LF/HF ratio did not change from pre‐injection to maximal Ver effect in dams (*P* = 0.738) or fetuses (*P* = 0.0701); the fetal value differed between maximal effect and recovery (*P* = 0.0366), whereas the corresponding dam comparison did not (*P* = 0.751).

### 3.5 Assessment of maternal and fetal electrical interference

Figure 9 shows representative recordings used to assess maternal cardiac electrical interference in fetal ECG channels. Figure 9a shows a control recording obtained in intentionally suboptimal earthing conditions. In these conditions, small maternal QRS‐related interference signals were visible in both fetal channels (red arrows) and were synchronized with maternal QRS waves (green numbers). These artefacts were much smaller than the fetal QRS complexes (blue and yellow numbers), indicating that maternal interference, when present, was identifiable and did not obscure fetal QRS detection. Figure 9b shows a representative recording obtained in the optimized earthing conditions used for formal experiments. In the full recording (Figure 9b1), maternal interference was barely detectable. Magnified views (Figure 9b2, b3) revealed only very small maternal QRS‐related deflections in the fetus A channel and no apparent maternal interference in the fetus B channel. These minor deflections could slightly alter local P wave (black arrows) or QRS (blue arrows) morphology but had minimal effect on fetal QRS identification or RR interval analysis.

**FIGURE 9:**
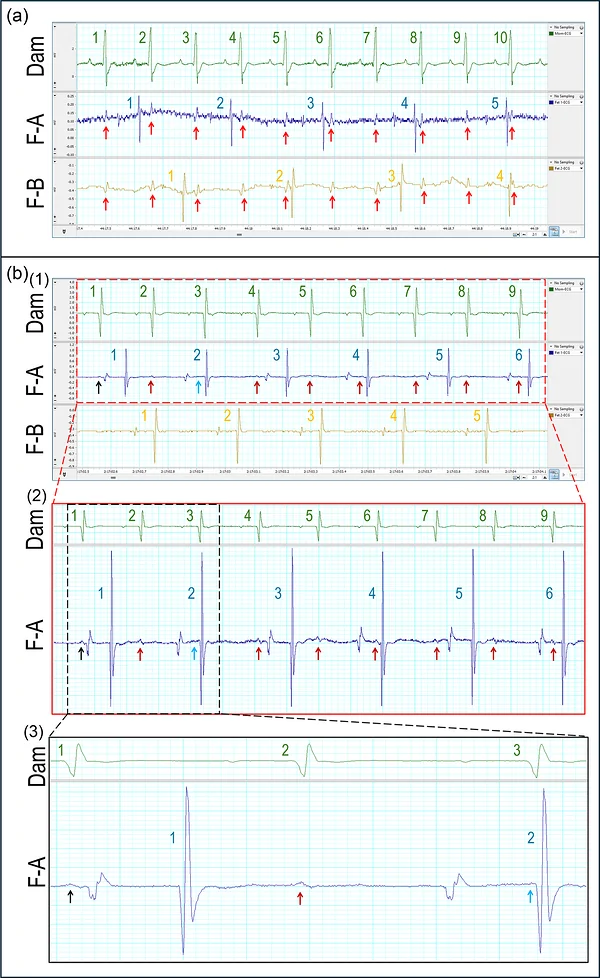
Assessment of maternal cardiac electrical interference in fetal ECG channels. (a) ECG obtained with intentionally suboptimal earthing, showing maternal QRS‐related interference in the fetal channels (red arrows). (b) ECG recording with optimized earthing used for formal experiments: (b1) full recording; (b2, b3) magnified regions indicated by the dotted rectangles in (b1). Black, dark red and blue arrows indicate small maternal QRS‐related deflections superimposed on the P‐wave region, baseline and QRS‐wave region of the fetus A ECG.

Figure 10 shows a control test evaluating whether addition of fetus A ECG recording channel altered the ECG signals recorded simultaneously from the dam and fetus B. No obvious change was identified in fetus B QRS morphology before (Pre) versus after (Post) activation of the fetus A recording channel. These observations suggest that adding the fetus A recording channel did not produce measurable interference in the fetus B or maternal ECG channels and support minimal cross‐fetal recording interference in the present experimental conditions.

**FIGURE 10:**
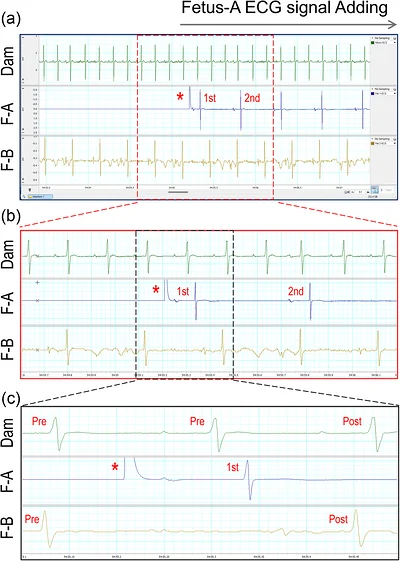
Effect of adding the fetus A ECG recording channel on simultaneous dam and fetus B ECG recordings. (a) Full recording during activation of the fetus A ECG channel. (b, c) Magnified regions indicated by the dotted rectangles in (a). The asterisk indicates the transient artefact generated when the fetus A preamplifier was turned on. 1st and 2nd indicate the first two QRS complexes detected from fetus  A after activation of the preamplifier. Pre and Post indicate dam and fetus B QRS complexes recorded before and after activation of the fetus A ECG recording channel.

## 4 DISCUSSION

The principal contribution of this Methods and Techniques study is a reproducible rat preparation that synchronizes three physiological data streams: continuous maternal arterial pressure, maternal ECG and direct ECG from two fetuses in the same pregnancy. The preparation remained stable for ≤5 h, supported interval and HRV analyses, and detected fetal cardiac responses during acute maternal pharmacological perturbation. We therefore view the method primarily as an acquisition and experimental‐control platform rather than as evidence, by itself, of maternal–fetal cardiac coupling.

Simultaneous acquisition is important physiologically because maternal haemodynamics and fetal cardiac activity can be aligned at beat‐to‐beat resolution. This makes it possible to quantify response latency and to apply established or emerging dynamic analyses to maternal and fetal signals. Prior mouse studies have demonstrated maternal–fetal ECG acquisition and changes in coupling‐related measures during autonomic blockade (Khandoker et al., 2018, 2022), and recent rat work has shown the value of time‐resolved autonomic modelling beyond conventional time‐averaged HRV measures (Candia‐Rivera et al., 2026). The present method is complementary to those analytical approaches because it supplies synchronized maternal BP and multi‐fetal ECG signals on which such analyses can be performed.

A second methodological strength is simultaneous recording from two fetuses. Earlier invasive maternal–fetal ECG studies established feasibility but generally focused on a single fetal channel (Kasahara et al., 2020; Khandoker et al., 2016, 2018, 2022). The dual‐fetal configuration permits two fetal responses to be observed during the same maternal perturbation and can support future analyses of fetus‐to‐fetus heterogeneity. Control recordings in Figures 9 and 10 indicate that, with optimized earthing and independent channels, maternal electrical contamination and cross‐fetal interference were small enough not to prevent fetal QRS identification and RR interval analysis.

Differences in waveform amplitude or morphology between fetus A and fetus B should be interpreted cautiously. Because each fetal ECG is obtained with a bipolar electrode pair inserted into a fetus through the uterine wall, the recorded waveform depends strongly on fetal position, fetal orientation, electrode angle and the electrical axis sampled by the electrode pair. Therefore, differences in morphology between fetuses do not by themselves indicate differences in cardiac physiology or signal quality. Regarding fetal position, we selected one accessible fetus from each uterine horn to maximize spatial separation and minimize lead/electrode interference. The position within a horn might influence signal amplitude and morphology because it changes access, electrode angle and proximity to surrounding fetal/maternal electrical fields. Future studies should examine whether fetal position within the horn affects signal quality.

The prolonged stability experiment showed that maternal arterial BP, maternal HR and fetal HR were maintained from the beginning to the end of a 5 h recording. This is useful for experiments requiring baseline characterization, sequential perturbations or time‐resolved analyses. Most applications, however, are likely to require substantially shorter windows; the 5 h experiment was intended to demonstrate an upper range of preparation stability rather than a required duration.

The ECG signals supported identification of P waves and QRS complexes and measurement of HR, RR interval and PR interval. Baseline differences between dams and fetuses and the lower fetal RMSSD, HF power and SDRR confirm that the recordings contain analysable electrophysiological and beat‐to‐beat timing information. These static differences are useful for method validation, but they should not be interpreted as demonstrating dynamic maternal–fetal interaction. Such interaction requires analyses that explicitly test time‐varying covariation, phase locking, directionality or response coupling (Candia‐Rivera & Chavez, 2025; Ducas et al., 2021; Khandoker et al., 2019; Piña‐Martínez et al., 2025; Steyde et al., 2026).

The pharmacological experiments further demonstrate system responsiveness. NA is a β‐adrenergic receptor agonist that induces peripheral vasoconstriction, leading to an acute increase in BP (McGrath & O'Brien, 1987), and Ver is a non‐dihydropyridine L‐type calcium channel blocker that inhibits calcium influx into cardiac pacemaker cells, cardiomyocytes and vascular smooth muscle, leading to bradycardia and hypotension (Pribil Pardun et al. et al., 2025). In the present study, NA produced a rapid maternal pressor response and bradycardia with a delayed fetal HR response, whereas Ver produced maternal hypotension/bradycardia and a different fetal response time course. These distinct temporal patterns show that the platform can resolve maternal and fetal changes during acute perturbation. They do not, however, identify the pathway linking the maternal intervention to the fetal response. Depending on the intervention, contributions might include transplacental drug transfer, altered uteroplacental perfusion, maternal autonomic reflexes and secondary neurohumoral mechanisms.

### 4.1 Limitations

Several limitations should be considered. First, this is an acute terminal preparation under isoflurane general anaesthesia, which alters autonomic and cardiovascular regulation; HRV findings therefore reflect anaesthetized physiology. Second, fetal electrode placement is invasive and technically demanding. Third, the study did not directly compare signal accuracy or sensitivity with Doppler ultrasound or non‐invasive abdominal ECG; our claims are therefore limited to feasibility, stability and the capabilities demonstrated within this preparation. Fourth, the concurrent maternal and fetal responses shown here do not constitute a formal analysis of maternal–fetal cardiac coupling. Dedicated dynamic analyses, such as phase‐coupling, cross‐sample entropy, time‐resolved autonomic modelling or other covariation methods, will be required for that purpose. Finally, only two fetuses per pregnancy were recorded, and those fetal observations share a maternal environment; future mechanistic studies should use statistical models that explicitly account for nesting within pregnancy.

These limitations also define the most useful future applications of the method. The synchronized signals can be used to test fetal autonomic maturation, maternal haemodynamic perturbations, pharmacological safety, placental or metabolic disease models and dynamic maternal–fetal cardiac relationships. Because the method records maternal arterial BP together with two direct fetal ECGs, it is particularly suited to studies that require temporal alignment of a controlled maternal physiological change with fetal electrophysiological responses.

## 5 CONCLUSION

We describe a reproducible platform for simultaneous maternal arterial BP, maternal ECG and dual‐fetal ECG acquisition in anaesthetized late‐gestation rats. The preparation supports prolonged beat‐to‐beat recording, ECG‐interval and HRV analyses, and acute pharmacological perturbation. Its key methodological advance is the integration of maternal haemodynamics with direct ECG from two fetuses within the same pregnancy.

The method is intended to complement, not replace, Doppler ultrasound, non‐invasive ECG or previously described invasive maternal–fetal ECG approaches. It does not by itself establish maternal–fetal coupling or the mechanism of fetal responses. Instead, it provides synchronized physiological signals and a standardized preparation that can support future dynamic, mechanistic and translational studies of maternal–fetal cardiovascular physiology. A screen recording of the monitor displaying maternal and fetal ECGs obtained using this technique is shown in video S1

## AUTHOR CONTRIBUTIONS

Lie Gao and Sarah Pribil Pardun designed the study. Lie Gao and Sarah Pribil Pardun performed the experiments, with assistance from Spencer Duff, Payton Ward and Auspy Li. The data were analysed by Lie Gao and Sarah Pribil Pardun. All authors contributed to manuscript preparation, approved the final version of the manuscript and agree to be accountable for all aspects of the work in ensuring that questions related to the accuracy or integrity of any part of the work are appropriately investigated and resolved. All persons designated as authors qualify for authorship, and all those who qualify for authorship are listed.

## CONFLICT OF INTEREST

None declared.

## GENERATIVE AI STATEMENT

The authors confirm that no generative AI tools were used in generating experimental data or performing statistical analyses.

## Supporting information




Supporting Information

Supporting Information

## Data Availability

The individual numerical data underlying the figures are provided as Supporting Information. The authors are willing to help and advise investigators who are interested in using these recording methods.
